# Identification of the PsEXP Gene Family and Functional Analysis of *PsEXPA4-1* During Flower Opening Process in Tree Peony (*Paeonia suffruticosa*)

**DOI:** 10.3390/genes17050586

**Published:** 2026-05-21

**Authors:** Jiayi Ying, Zhishuang Wang, Yinuo Shen, Yangdeng Lin, Yao Wang, Congying Zhu, Yiyang Xu, Luanfang Lin, Xiangtao Zhu, Xia Chen, Qianqian Wang

**Affiliations:** 1 College of Jiyang, Zhejiang A&F University, Zhuji 311800, China; yjyhfg@163.com (J.Y.); 17398081659@163.com (Z.W.); 18968086099@163.com (Y.S.); 13566839539@163.com (Y.L.); jiuzhong7986@163.com (Y.W.); 15712627849@163.com (C.Z.); 18757688332@163.com (Y.X.); zhuxt@zafu.edu.cn (X.Z.); 2 Ningde Agricultural Science Research Institute, Ningde 352000, China; linluanfang@126.com

**Keywords:** *Paeonia suffruticosa*, flower opening, gibberellin, *PsEXP4-1*, *PsbHLH25*

## Abstract

Background/Objectives: Tree peony (*Paeonia suffruticosa*) has a short flowering period, and its regulatory mechanisms remain poorly understood. These limitations have severely constrained its industrial development. Expansins (EXPs) are key proteins that mediate cell wall loosening associated with petal expansion, yet they remain uncharacterized in tree peony. Methods: This study utilized gene family identification, key gene screening and functional research, as well as regulatory network analysis to reveal the role of the EXP family in the regulation of flower opening. Results: This study presents the first genome-wide identification of 36 *PsEXP* genes in tree peony, classifying them into four evolutionarily conserved subfamilies (PsEXPA, PsEXPB, PsEXLA, and PsEXLB). Promoter analysis revealed that 28 out of 36 *PsEXP* promoters contain gibberellin (GA)-responsive elements. Exogenous GA_3_ treatment significantly promoted flower opening and upregulated eight *PsEXPs*, with *PsEXPA4-1* showing the highest expression level and promoter GA-responsive element abundance. Subcellular localization confirmed that PsEXPA4-1 was targeted to the cell wall. Overexpression of *PsEXPA4-1* in *Arabidopsis* led to early flowering and enlarged petals, indicating that *PsEXPA4-1* had the potential to promote cell expansion, consistent with its proposed role in tree peony flower opening. Mechanistically, we identified a bHLH transcription factor, PsbHLH25, whose expression was induced by GA. Y1H and dual-luciferase assays indicated that PsbHLH25 can bind to the *PsEXPA4-1* promoter. Conclusions: This study systematically characterized the EXP gene family in tree peony, revealed *PsEXPA4-1* as a key effector downstream of GA promoting flower opening, and discovered *PsbHLH25* as a transcriptional activator linking GA signaling to *PsEXPA4-1*. These findings provided important insights into GA-mediated floral opening and genetic resources for understanding the molecular mechanisms and enabling precise flowering time regulation in tree peony.

## 1. Introduction

Floral display is a key horticultural trait in ornamental plants, possessing significant economic and esthetic value [1]. The process of flower formation is complex, typically encompassing the following three stages: floral transition, floral bud development, and flower opening [2,3]. Among these, the flower opening process can directly influence flowering time and is crucial in the regulation of plant flowering phenology [4]. However, in tree peony (*Paeonia suffruticosa*), a high-value ornamental species with a notoriously short flowering period, the molecular mechanisms regulating flower opening remain largely unexplored.

Flower opening progresses through bud swelling, anthesis initiation, petal bending, and full unfolding, driven by petal expansion and movement [5,6]. This process proceeds through distinct developmental phases. The division and expansion of petal epidermal cells serve as the primary drivers of petal movement, ultimately determining whether flowers open normally [7,8]. The enlargement of petal epidermal cells relies on coordinated changes in cell wall metabolism, cell turgor pressure, and cytoskeleton reorganization [7]. However, the cell wall is typically rigid, maintaining cell shape stability through a robust fibrous network [9]. Therefore, during flower opening, plants selectively loosen the cell wall network or matrix to allow accommodation to expansive forces generated by turgor pressure [9]. Petal cell expansion requires coordinated cell wall loosening. Enzymes associated with cell wall loosening include pectinases, endo-1,4-β-D-glucanases, xyloglucan endotransglycosylases/hydrolases (XTHs), and expansins (EXPs), among which EXP proteins play a significant role [10,11]. Among these, EXPs are unique because they directly mediate acid-induced cell wall extension without hydrolytic activity, making them key regulators of rapid cell expansion during processes such as flower opening [8,12].

Based on differences in gene structure and amino acid sequence composition, plant EXP proteins are categorized into the following four subfamilies: EXPA (α-expansin), EXPB (β-expansin), EXLA (expansin-like A), and EXLB (expansin-like B) [8]. EXPA and EXPB are the two primary expansin subtypes in plants, being both numerically and functionally predominant, although their distribution varies considerably among different species [12]. Functional studies have revealed that EXPs influence cellulose synthesis or deposition during petal cell wall assembly, determining petal cell size and thereby affecting the flower opening process [5]. In various plants such as *Petunia hybrida* [13], *Dianthus caryophyllus* [14], *Nelumbo nucifera* [15], and *Rosa chinensis* [16], EXP proteins have been implicated in regulating flower opening. Transformation of *Petunia* with an antisense expression vector for *PhEXP1* resulted in transgenic plants with significantly smaller corolla areas compared to controls, indicating that *PhEXP1* directly regulates cell wall metabolism in petunias [17]. Another expansin gene in petunia, *PhEXPA1*, has also been confirmed to participate in regulating cell wall metabolism [13]. The unfolding state of carnation petals during flower opening is closely correlated with the expression levels of *DcEXPA1* and *DcEXPA2* [14]. In roses, the transcriptional levels of *EXPs* increased significantly during petal growth, and the rate of petal area expansion correlated with the expression level of *RhEXPA7* [16]. Similarly, the expression level of *GgEXPA1* was associated with changes in petal area in *Gladiolus grandiflorus* [18]. During vegetative growth, EXP proteins are also known to promote leaf expansion and stem elongation, although the present study focuses on their role in flower opening [19,20].

Further research has revealed that *EXPs* not only play a significant role in the development of plant organs and tissues, but their expression is also influenced by plant hormones [5,7]. Gibberellic acid (GA), an important class of hormones in plant growth, development, and flowering regulation, has been extensively studied in the context of flower opening [21]. Research on rapeseed (*Brassica campestris*), cotton (*Gossypium hirsutum*), and *Arabidopsis* has shown that GA promotes *EXP* expression to regulate stem elongation, epidermal cell differentiation, and flower development [22,23,24]. In addition, transcriptional regulators such as DELLA and NAC proteins have been identified as upstream modulators of *EXP* genes in GA signaling pathways [24,25]. However, in tree peony, whether and how GA signaling regulates *EXP* expression during flower opening remains unknown.

Tree peony, renowned as one of China’s top ten traditional famous flowers, has become a significant commodity in the Lunar New Year flower market due to its high ornamental value and profound cultural significance [26,27]. However, the natural flowering period of tree peony predominantly occurs between March and May, with individual plants having a short blooming duration [28]. Precise flowering regulation is therefore required to meet market demand for the Spring Festival. Current flowering regulation for Lunar New Year tree peonies primarily relies on forcing cultivation in autumn and winter, encompassing the following two stages: dormancy release of mixed buds and flower opening [26]. Research and practice have mainly focused on the dormancy release stage, while the regulatory mechanisms governing the subsequent flower opening process remain inadequately understood [29]. Under forcing cultivation conditions, tree peonies still require a prolonged period of 60–70 days, involving multiple developmental stages, from dormancy release to full flower opening [30]. This leads to challenges in Lunar New Year peony production, including time and labor intensiveness, unpredictable flowering times, and even forcing failure [31]. Our previous research on the flowering process of peonies indicated that exogenous GA_3_ plays a significant role in flower development regulation, and the optimal treatment concentration is 800 mg/L [28]. However, in tree peony, the downstream effectors of the GA response and whether the EXP gene family can respond to GA signals to regulate the flowering process remain largely unknown.

To investigate whether GA signaling can promote flower opening in tree peony by upregulating specific *EXP* genes, this study utilizes the important Lunar New Year peony cultivar “Lu Hehong” (*P. suffruticosa* “Lu Hehong”) as experimental material. Based on genomic data [32], we identified and analyzed members of the EXP gene family in tree peony and examined their expression patterns in response to different concentrations of exogenous GA_3_. The key *EXP* members responsive to GA signaling that promote flowering in tree peony were screened. Additionally, *PsEXPA4-1* showing the highest expression and greatest promoter GA-element abundance was selected for detailed functional analysis. Through extensive bioinformatics analysis and functional validation, we aimed to elucidate the roles of *PsEXPA4-1* in mediating GA_3_ signaling to promote the flower opening process. The outcomes of this project are expected to further refine the molecular network of GA signaling in regulating tree peony flower opening and provide a critical theoretical foundation for advancing flowering regulation techniques and promoting the development of the Lunar New Year peony industry.

## 2. Materials and Methods

### 2.1. Plant Materials

The experimental materials were perennial potted plants of “Luhehong”, cultivated at the Tree Peony Experimental Base of Jiyang College, Zhejiang A&F University. All plants were six years old and showed uniform growth and developmental status at the start of the experiment. Samples from different plant tissues and various developmental stages were collected, with each sample weighing 0.5 g and processed in triplicate. The samples were rapidly frozen in liquid nitrogen and subsequently stored at −80 °C for further use.

### 2.2. Identification and Structural Analysis of the Members from the PsEXP Gene Family

The published genomic and annotation files of “Fengdan” were downloaded from the relevant data platform (https://db.cngb.org/search/project/CNP0000281/, accessed on 20 April 2025) [32]. Combined with the full-length transcriptome data obtained from sequencing “Luhehong”, the members of the PsEXP gene family in tree peony were screened. The PsEXP gene family was identified using the following criteria. First, the hidden Markov model (HMM) profile of the expansin domain (PF03330) was downloaded from the Pfam database (https://pfam.xfam.org/, accessed on 18 June 2025). Second, HMMER v3.0 was used to search against the tree peony genome with an E-value threshold of 1 × 10^−5^. Third, candidate sequences were further validated by confirming the presence of the conserved DPBB_1 domain (PF03330) and the pollen_allergen_1 domain (PF01357) using SMART (http://smart.embl-heidelberg.de/, accessed on 18 June 2025) and NCBI-CDD (https://www.ncbi.nlm.nih.gov/Structure/cdd/wrpsb.cgi, accessed on 18 June 2025). Sequences lacking either domain were excluded. The full set of EXP homologous sequences from the *Arabidopsis* genome was downloaded from the TAIR11 database (https://www.arabidopsis.org/, accessed on 20 June 2025), from which candidate AtEXP protein sequences were selected. The identified PsEXP protein sequences from tree peony and the AtEXP protein sequences from *Arabidopsis* were aligned using MEGA 11 software. A phylogenetic tree was constructed using the Neighbor-Joining (NJ) method, with branch support assessed through Bootstrap testing (1000 replicates). The phylogenetic tree was subsequently annotated and refined using the online iTOL v3.0 tool (https://itol.embl.de/, accessed on 25 June 2025). The *PsEXP* genes were systematically named based on their transcriptomic characteristics and phylogenetic relationships with 34 *Arabidopsis* homologs. First, all genes were assigned to subfamilies (EXPA, EXPB, EXLA, and EXLB) based on phylogenetic clustering with *Arabidopsis* AtEXP homologs. Second, within each subfamily, genes were numbered sequentially based on their genetic relationship with *Arabidopsis* and chromosomal position from Chr01 to Chr05. Third, for genes that are highly similar and form a clade with an *Arabidopsis* homolog, a hyphenated suffix (e.g., −1, −2) was added to indicate multiple members within the same clade. Genes that could not be mapped to chromosomes were numbered after the chromosome-mapped ones. Based on the obtained tree peony genomic and annotation files, the exon-intron structures were determined using the online software GSDS 2.0 (http://gsds.cbi.pku.edu.cn, accessed on 30 June 2025).

### 2.3. Chromosomal Localization and Synteny Analysis of the PsEXP Gene Family

The annotation information of the EXP gene family in tree peony was extracted using TBtools v2.136. Chromosomal localization was visualized employing the “Gene Location Visualize from GTF/GFF” module. Synteny analysis was subsequently performed using the “One Step MCScanX” module in TBtools.

### 2.4. Cis-Acting Element Analysis of the PsEXP Gene Family

To identify cis-regulatory elements in the upstream regions of *PsEXPs*, 2000 bp sequences upstream of the start codon of each *PsEXP* were extracted from the tree peony genome and annotation files. These sequences were subsequently submitted to the PlantCARE 2.0 database (http://bioinformatics.psb.ugent.be/webtools/plantcare/html/, accessed on 25 June 2025) for prediction, analysis, and screening of cis-acting elements.

### 2.5. Exogenous GA_3_ Treatment

After natural dormancy release, the plants were transferred to the greenhouse facility of the experimental base in December 2024 for subsequent management. The greenhouse environment was maintained with an average daytime temperature of 25 °C and a photoperiod of 14 h of light followed by 10 h of darkness. A total of 30 potted peony plants with consistent growth status, each bearing 6–10 floral buds, were selected and evenly divided into two groups. Based on previous study [28], we tested different GA_3_ concentrations on flower opening process in tree peony and 800 mg/L was selected as the optimal concentration for promoting flower opening without adverse effects. From 9 to 11 January 2025, floral buds were treated daily for three consecutive days with either 800 mg/L GA_3_ solution (containing 0.1% phosphoric acid and 0.025% Triton X-100, Aladdin Biochemical Technology Co., Ltd., Shanghai, China) or control solution (same buffer without GA_3_). Floral bud samples (n = 3 per group) were collected on days 0, 10, 20, 30, and 40, immediately frozen in liquid nitrogen, and stored at −80 °C.

### 2.6. RNA Extraction for Quantitative Real-Time PCR Analysis

Total RNA was extracted using an RNAprep Pure Plant Kit (TianGen, Beijing, China, #DP441), and its quality was evaluated using a nucleic acid analyzer (Implen Company, Munich, Germany) and agarose gel electrophoresis. First-strand cDNA was synthesized using the PrimeScriptTM RT kit (TaKaRa, Dalian, China, #639506). The *PsACT* of “Luhehong” was used as the internal reference gene for quantitative analysis [27]. All the primer sequences for *PsEXPs* and *PsACT* are listed in Supplementary Table S1. Quantitative reverse transcription polymerase chain reaction (qRT-PCR) was performed using the LightCycler^®^ 480 II system (Roche, Basel, Switzerland). The reaction mixture (total volume of 20 µL) consisted of 10 µL of enzyme mix, 2 µL of cDNA, 0.8 µL each (10 µmol/L) of forward and reverse primers, and 6.4 µL of ddH_2_O. The amplification program was set as follows: initial denaturation at 95 °C for 30 s, followed by 40 cycles of denaturation at 95 °C for 5 s and annealing/extension at 60 °C for 30 s. A final dissociation step was performed at 95 °C for 5 s, 60 °C for 1 min, and 95 °C for 15 s. All qRT-PCR experiments were conducted with three independent biological replicates. For each biological replicate, three technical replicates were performed and averaged before statistical analysis. The 2^−ΔΔCT^ method was used to determine the relevant expression.

### 2.7. Promoter Cloning and Activity Detection

Genomic DNA was isolated from buds of the cultivar “Lu Hehong” employing a commercial DNA extraction kit (Vazyme, Nanjing, China, #DC112-02) in accordance with the manufacturer’s protocol. Primer pairs specific to the promoters of *PsEXPs* were designed with Primer Premier software (version 6.0) and subsequently validated by conventional PCR amplification. To investigate the transcriptional regulation of *PsEXPs* by exogenous gibberellin (GA_3_) signaling, their promoter fragments were directionally cloned into the plant binary vector pCAMBIA1300-GUS. The resulting recombinant plasmids, proPsEXPs::GUS, were independently introduced into *Agrobacterium tumefaciens* strain GV3101 via electroporation. Stable tree peony callus lines were generated following a previously established protocol. Subsequently, tree peony callus (homologous, closer to native context) and tobacco leaves (heterologous, allowing rapid transformation) were transformed with the respective promoter-GUS constructs via an optimized vacuum infiltration method. For GA_3_ treatment assays, transformed materials were incubated in darkness at 25 °C for 24 h prior to exposure to liquid medium containing either 0 mg/L (mock control) or 800 mg/L exogenous GA_3_. Following 6 h of treatment, samples were harvested for histochemical β-glucuronidase (GUS) staining and quantitative fluorometric GUS activity assays, performed according to the manufacturer’s instructions using a commercial kit (Coolaber, Beijing, China, #SL7160 and #SL7161). All promoter activity assays were performed using three independent biological replicates. For each biological replicate, three technical replicates were measured and averaged.

### 2.8. Sequence and Phylogenetic Analysis

Full-length open reading frames of *PsEXPA4-1* were amplified using the first-strand cDNA of the “Lu Hehong” flower buds. Sequences from *Carica papaya* (*CpEXPA8*, XP_021904225.1), *Morus notabilis* (*MnEXPA4*, XP_010108480.1), *Quillaja saponaria* (*QsEXPA4*, KAJ7964314.1), *Fagus crenata* (*FcEXPA8*, GMY10440.1), *Spatholobus suberectus* (*SsEXPA8*, TKY56599.1), *Sesamum indicum* (*SiEXPA4*, XP_011089527.1) and *Actinidia eriantha* (*AeEXPA4*, XP_057513338.1) were downloaded from GenBank. Sequence alignment was performed using DNAMAN (version 7.0). The phylogenetic tree was constructed using MEGA 11, with the Neighbor-Joining (NJ) method and branch support assessed through Bootstrap testing (1000 replicates). The phylogenetic tree was subsequently annotated and refined using the online ITOL v3.0 tool (https://itol.embl.de/, accessed on 10 December 2025).

### 2.9. Subcellular Localization

The coding sequence (CDS) of *PsEXPA4-1* was amplified and subsequently cloned into the 35S::GFP vector (conferring kanamycin resistance). The resulting recombinant plasmid was isolated and then introduced into *A*. *tumefaciens* strain GV3101 via the heat-shock transformation method. The plasmid was transformed into onion (*Allium cepa*) epidermal cells via agroinfiltration, a well-established heterologous system for subcellular localization. The localization of the PsEXPA4-1-GFP fusion protein was observed using confocal microscopy. Subcellular localization of the PsEXPA4-1-GFP fusion protein was observed and imaged using a high-resolution confocal laser scanning microscope (TCS SP8 MP; Leica Microsystems GmbH, Wetzlar, Germany).

### 2.10. Genetic Transformation of Arabidopsis

To investigate the biological function of *PsEXP4-1*, the recombinant 35S::PsEXP4-1-GFP plasmid was transferred into *A. tumefaciens* strain GV3101. Wild-type *Arabidopsis* thaliana (Col-0) plants were transformed using the floral dip method. Transgenic seeds were selected on Murashige and Skoog (MS) medium supplemented with 50 mg/L kanamycin. Putative transgenic plants were confirmed by PCR analysis. Homozygous T_3_ generation plants were used for phenotypic characterization and expression analysis. For phenotyping, flowering time and the number of rosette leaves at bolting were recorded for 15 individual plants per genotype. The dimensions (length and width) of the largest rosette leaves from 15 plants per line were measured. Petal size was quantified using fully opened flowers from five independent plants per genotype, with three flowers per plant. For each flower, the length and width of four petals were measured, and average values were calculated per flower.

### 2.11. Yeast One-Hybrid Assay

The PsEXPA4-1 promoter fragment used as bait in the yeast one-hybrid assay was 2000 bp in length, spanning from −2000 to −1 relative to the ATG start codon. The promoter fragment was cloned into the pHis2 vector. The resulting construct was introduced into yeast strain Y187 to determine the optimal inhibitory concentration of 3-amino-1,2,4-triazole (3-AT). Subsequently, a cDNA library was constructed using total RNA extracted from “Luhehong” flower buds at different developmental stages. The library quality was assessed, showing a titer of >1 × 10^6^ CFU/mL and an average insert size of approximately 1.0 kb. The library was screened under the established selection condition (40 mM 3-AT) to identify proteins interacting with the PsEXPA4-1 promoter. Protein–DNA interactions were validated using the MatchmakerTM Gold Yeast One-Hybrid System (Coolaber, Beijing, China; #YH1011).

### 2.12. Dual-Luciferase Reporter Assay

The transcriptional regulation of *PsEXP4-1* by PsbHLH25 was examined using the Dual-Glo^®^ Luciferase Assay System (Promega, Madison, WI, USA). The coding sequence of *PsbHLH25* was fused to the 35S promoter in the pORER4-35S-GFP vector, while the *PsEXP4-1* promoter was inserted upstream of the luciferase gene in pGreen0800II-LUC. Both constructs, together with the pSoup helper plasmid, were co-transformed into *A. tumefaciens* GV3101. Bacterial suspensions (OD_600_ = 0.6) were prepared and incubated at room temperature for 3 h before infiltration.

Equal volumes of PsbHLH25-GFP and *proPsEXPA4-1*-LUC agrobacterial suspensions were mixed and infiltrated into leaves of *Nicotiana benthamiana* plants. After infiltration, plants were kept in darkness at 23 °C for 24 h and then transferred to a growth chamber with a 14 h light/10 h dark photoperiod at 23 °C for 48 h. Luciferase activity was visualized using an in vivo plant imaging system (Tanon 700, Shanghai, China) and quantitatively measured with a GLOMAX^®^ multi-function detection system (Promega, Madison, WI, USA) using the Dual-Luciferase^®^ Reporter Assay Kit (Promega, E1910). The pGreen0800II-LUC vector contains both LUC driven by the PsEXPA4-1 promoter and REN as an internal control. Relative LUC activity was calculated as the LUC/REN ratio to normalize for transformation efficiency. Three independent biological replicates (each with three technical replicates) were performed for the dual-luciferase assay.

### 2.13. Statistical Analysis

All statistical analyses were conducted using SPSS software (version 16.0; SPSS Inc., Chicago, IL, USA). Data are expressed as means ± standard deviation (SD) from three independent biological replicates. Student’s *t*-test was used for comparisons between two groups. One-way ANOVA followed by Duncan’s multiple range test was used for comparisons involving more than two groups. Normality and homogeneity of variances were checked using Shapiro–Wilk and Levene’s tests, respectively, before parametric analyses. A threshold of *p* < 0.05 was considered statistically significant.

## 3. Results

### 3.1. Identification and Structural Analysis of the PsEXP Gene Family

**Figure 1:**
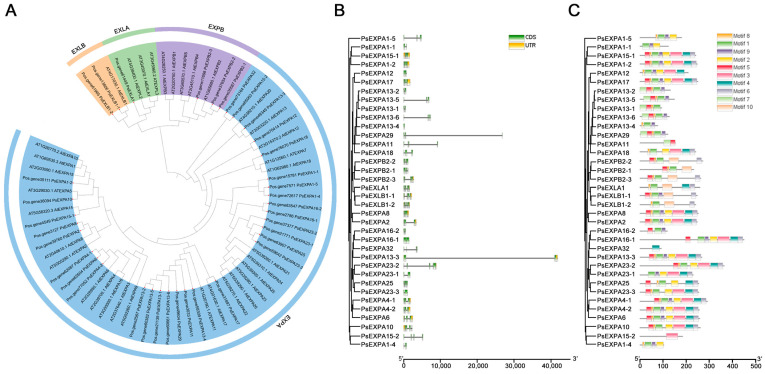
Identification and structural analysis of the PsEXP proteins. (**A**) Phylogenetic relationship of PsEXP proteins. Different background colors represent four different groups of the PsEXP proteins. The PsEXP proteins were marked by red points. The At and Ps represent *A.thaliana* and *P. suffruticosa*, respectively. (**B**) Structural analysis of the PsEXP proteins. The black lines represent introns, and the color boxes represent the coding sequences. The horizontal value represents gene length from 5′ to 3′. (**C**) Conserved motif analysis of the PsEXP proteins. Different motif numbers were marked by different color boxes. The horizontal value represents gene length from 5′ to 3′.

A total of 36 *PsEXP* genes were identified from the tree peony genome. Phylogenetic analysis further revealed that the PsEXP gene family in tree peony, along with its homologous genes in *Arabidopsis*, can be classified into the following four major subfamilies: EXPA, EXPB, EXPLA, and EXPLB. These four subfamilies are named PsEXPA, PsEXPB, PsEXLA, and PsEXLB, with 30, three, one, and two members, respectively (Figure 1A). Gene structure analysis revealed significant variation in sequence length and exon–intron distribution among the 36 identified *PsEXP* genes. Among these, only 15 *PsEXP* genes contained untranslated regions (UTRs), with *PsEXPA13-2* possessing the longest sequence and *PsEXPA13-4* exhibiting the shortest (Figure 1B). Analysis of motifs revealed that 10 conserved motifs were identified in the encoded protein products using the MEME suite. The majority of PsEXP proteins contain Motif1, Motif3, Motif5, and Motif6, indicating higher conservation of these four motifs within the PsEXP family of tree peony (Figure 1C). Based on domain annotation, these motifs map to the conserved DPBB_1 and pollen_allergen_1 domains, which are characteristic of expansin proteins and required for their activity. These four motifs suggested that they may be essential for expansin function, potentially related to cell wall binding and cell wall loosening activity. Notably, *PsEXPA32* and *PsEXPA15-2* contained only one conserved motif, which may indicate divergent evolution, potential truncation, or annotation artifacts. Further experimental validation is needed.

### 3.2. Chromosomal Localization and Synteny Analysis of the PsEXP Gene Family

**Figure 2:**
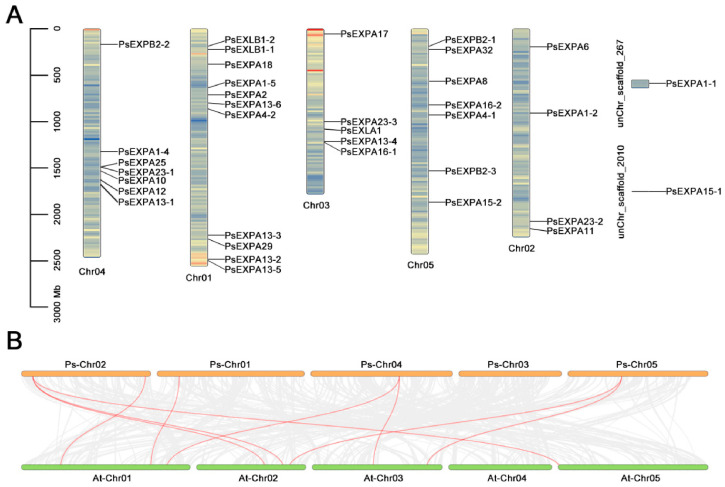
Chromosomal localization and synteny analysis of the EXP proteins. (**A**) Chromosomal localization of PsEXP proteins. The vertical value represented the length from different chromosomal. (**B**) The synteny analysis of the EXP proteins between tree peony and *Arabidopsis*. The At and Ps represented tree peony and *Arabidopsis*, respectively.

In the PsEXP gene family, with the exception of *PsEXPA1-1* and *PsEXPA15-1*, which were not mapped to the assembled chromosomes possibly due to incomplete genome assembly or their location on unplaced scaffolds, the remaining proteins were distributed across Chr01–05 (Figure 2A). Chr01 contained the highest number of PsEXP proteins (11 in total), followed by Chr04 and05, each harboring seven PsEXP proteins, while Chr03 contained five genes. Chr02 carried four PsEXP proteins. To further investigate the evolutionary relationships of PsEXP proteins among different species, a synteny analysis was conducted between tree peony and *Arabidopsis* (Figure 2B, Supplementary Table S2). The results revealed that five PsEXP proteins located on five chromosomes of tree peony exhibited syntenic relationships with nine AtEXP proteins distributed across five chromosomes of *Arabidopsis,* forming five syntenic gene pairs. These conserved syntenic blocks indicate that certain *EXP* genes have evolved under purifying selection and may retain similar functions across eudicots. This suggests that certain EXP proteins may share conserved functions between tree peony and *Arabidopsis*.

### 3.3. Promoter Analysis of PsEXP Genes

**Figure 3:**
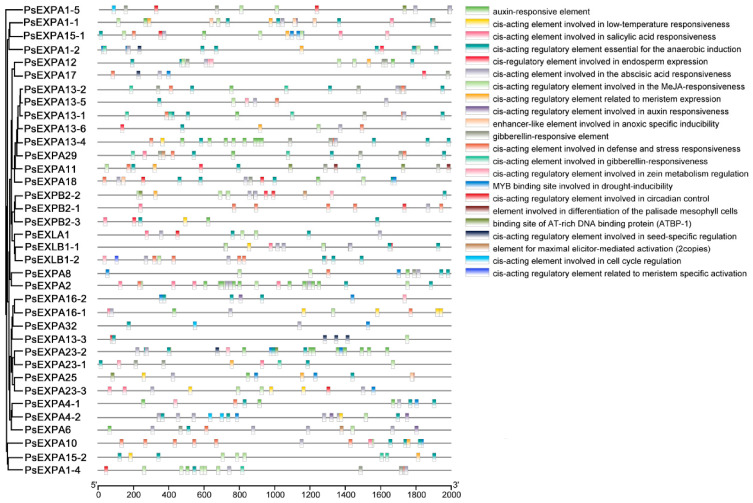
The cis-regulatory element (CRE) analysis of PsEXP gene promoters. The black lines represent the 2 kb promoter regions, and the different color boxes correspond with the different kinds of CREs. The horizontal values represent the promoter length.

To investigate the distribution of cis-acting elements in the promoters of different *PsEXP* genes, we extracted the 2000 bp sequences upstream of the ATG start codon as putative promoter regions based on the genomic data. The distribution of cis-acting elements within these promoter sequences was analyzed using the online tool PlantCare 2.0. The results revealed that the promoters of various *PsEXP* genes contain abundant hormone-responsive elements and transcription factor binding sites (Figure 3). Notably, gibberellin (GA)-responsive elements were identified in 28 out of 36 *PsEXP* promoters, suggesting a potential link to GA regulation. Although promoter cis-element analysis alone does not constitute strong functional evidence, it provides a useful basis for hypothesizing regulatory relationships.

### 3.4. Expression Patterns of PsEXP Genes Under Different GA Treatments

**Figure 4:**
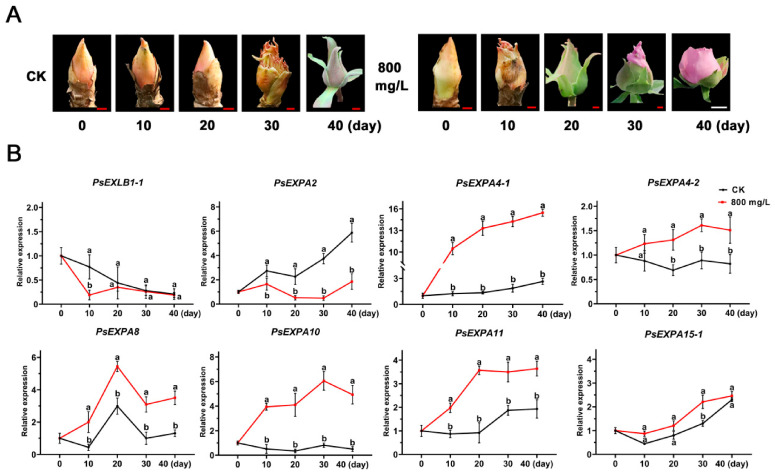
The expression patterns of *PsEXPs* under GA treatments. (**A**) Phenotypic changes under exogenous GA treatment. Red scale = 0.5 cm; white scale = 1 cm. CK: control (water treatment); GA: 800 mg/L GA_3_ treatment. (**B**) Expression analysis of *PsEXPs* under different treatment conditions. CK: control; GA: GA_3_ treatment. Different letters indicate significant differences among genes within the same treatment group, mean ± SD, n = 3, *p* < 0.05.

According to our previous research [28], plants were treated with 800 mg/L exogenous GA, and the expression patterns of *PsEXP* genes were analyzed. Consistent with earlier observations, GA treatment markedly promoted flower opening (Figure 4A). Expression analysis revealed that within the entire EXP gene family, 21 members showed undetectable expression levels under the tested conditions, likely because their transcript abundance was below the detection limit of qRT-PCR. Seven members exhibited no significant differential expression, and eight members were significantly upregulated in response to exogenous GA (Figure 4B, Supplementary Figure S1). Among these, the induction of *PsEXPA2*, *PsEXPA4-1*, *PsEXPA10*, and *PsEXPA11* showed higher levels. *PsEXPA4-1* displayed the highest expression level among all induced genes. Given the abundance of GA-responsive cis-elements in its promoter region, we propose that *PsEXPA4-1* may play a critical role in mediating GA-induced flower opening in tree peony.

### 3.5. GA Sensitivity of PsEXP Promoters and Validation of Candidate Genes

**Figure 5:**
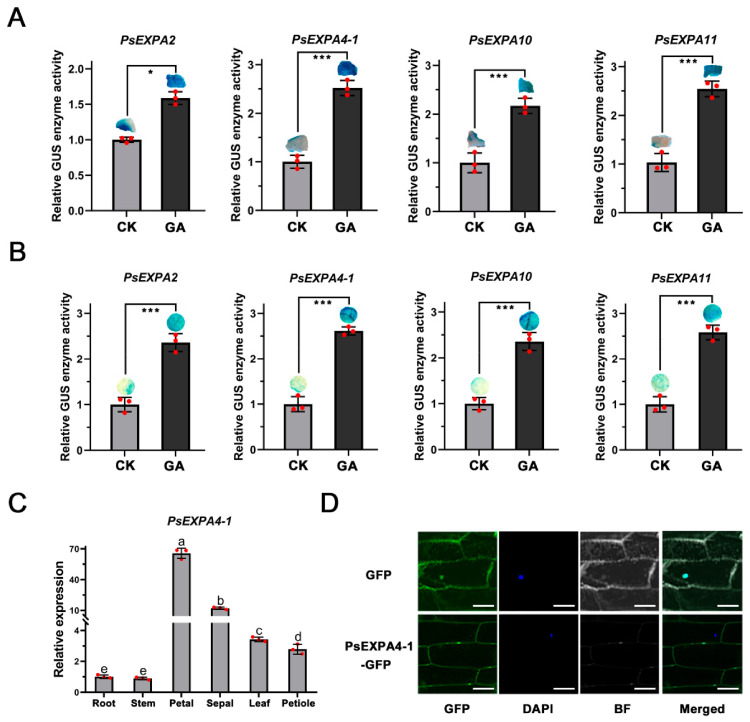
The promoter sensitivity analysis of *PsEXPs* under different treatments. (**A**) The promoter sensitivity analysis of *PsEXPs* to exogenous GA signal in tree peony callus. Means ± SD, n = 3, * *p* < 0.05, *** *p* < 0.001. (**B**) The promoter sensitivity analysis of *PsEXPs* to exogenous GA signal in tobacco leaves. Means ± SD, n = 3, *** *p* < 0.001. (**C**) The expression pattern analysis of *PsEXPA4-1* in different tissues. Different letters indicated significant differences, Student’s *t* test, *p* < 0.05, means ± SD, n = 3. (**D**) Subcellular localization of PsEXPA4-1 protein in onion epidermal cells. GFP: fluorescence signal; DAPI: nuclear localization; BF: bright field; Merged: merge images. Scale bar: 100 um.

To further validate whether the expressions of the candidate genes *PsEXPA2*, *PsEXPA4-1*, *PsEXPA10*, and *PsEXPA11* were directly induced by exogenous GA signaling, we assessed the activity of their promoters, both tree peony callus and tobacco leaves. The results demonstrated that exogenous GA significantly enhanced the promoter activity of these genes in both experimental systems (Figure 5A,B). Based on integrated analysis of promoter cis-elements and expression patterns, *PsEXPA4-1* was selected for further investigation. Sequence and phylogenetic analysis confirmed that PsEXPA4-1 contains the conserved expansin domains (DPBB with six conserved cysteine residues and pollen_allergen) [8] and clusters with EXPA homologs from *C. papaya* and *F. crenata* (Supplementary Figure S2A,B). Tissue-specific expression analysis revealed that *PsEXPA4-1* was predominantly expressed in petals, with lower expression in sepals (Figure 5C). Subcellular localization experiments indicated that the EXPA4-1 protein is primarily localized to the cell wall (Figure 5D).

### 3.6. Function Analysis of PsEXPA4-1

**Figure 6:**
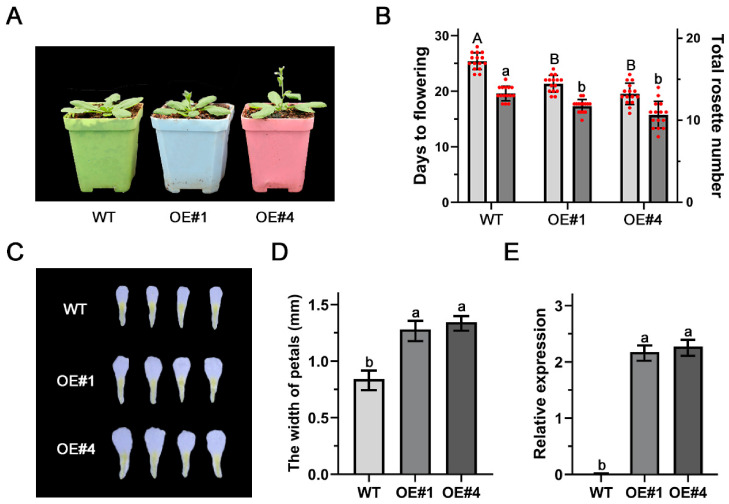
The function analysis of *PsEXPA4-1* in *Arabidopsis*. (**A**) Phenotypic analyses of transgenic plants *PsEXPA4-1*. (**B**) Analysis of flowering time and rosette leaves number in transgenic plants. Uppercase letters indicated the flowering time. Lowercase letters indicated the rosette leaves number. Means ± SD, n = 15, *p* < 0.05. (**C**) Phenotypic analyses of petals from transgenic plants. (**D**) The width analysis of the petals in transgenic plants. Different letters indicated significant differences, Student’s *t* test, *p* < 0.05. Means ± SD, n = 3. (**E**) Expression levels analysis of *PsEXPA4-1* in transgenic plants. Different letters indicated significant differences, Student’s *t* test, *p* < 0.05. Means ± SD, n = 3.

To further confirm the function of *PsEXPA4-1* in flower opening regulation, it was overexpressed in *Arabidopsis*. A total of 10 independent transgenic lines were obtained and confirmed by PCR. Three independent homozygous T_3_ lines (OE-3, OE-6, and OE-9) showing high *PsEXPA4-1* expression were selected for further phenotypic analysis. The results showed that overexpression of *PsEXPA4-1* significantly accelerated flowering compared to WT plants (Figure 6A). Analyses of rosette leaf number and flowering time indicated that *PsEXPA4-1* overexpression reduced rosette leaf number and promoted earlier flowering in *Arabidopsis* (Figure 6B). Statistical analysis of petal size across genotypes showed that the petal width of overexpressed lines was significantly greater than that of WT plants, being 0.42 mm and 0.48 mm larger than the control group respectively (Figure 6C,D). However, there was no significant difference in petal length (Supplementary Figure S3). Furthermore, expression analysis confirmed that *PsEXPA4-1* transcript levels were markedly elevated in overexpression lines, suggesting that enhanced *PsEXPA4-1* expression leads to earlier flowering and enlarged petals in *Arabidopsis* (Figure 6E).

### 3.7. Verification of the Combination of PsEXPA4-1 and PsbHLH25

**Figure 7:**
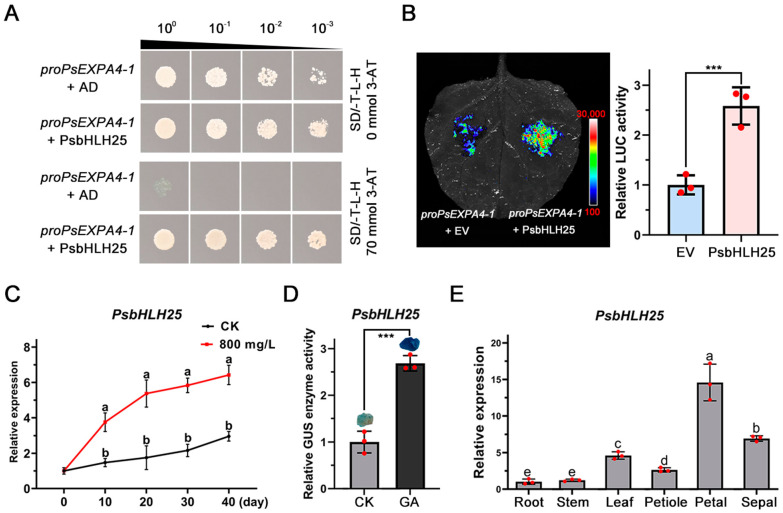
Binding analysis between PsbHLH25 protein and *PsEXPA4-1* promoter. (**A**) Yeast single hybridization analysis between PsbHLH25 protein and *PsEXPA4-1* promoter. (**B**) Dual-luciferase reporter analysis between PsbHLH25 protein and *PsEXPA4-1*. Ev: 35S::GFP empty. Means ± SD, n = 3, *** *p* < 0.001. (**C**) Analysis of expression patterns of *PsbHLH25* under different GA_3_ treatments. Different letters indicated significant differences, Student’s *t* test, *p* < 0.05, means ± SD, n = 3. (**D**) The promoter sensitivity analysis of *PsbHLH25* to exogenous GA signal in tree peony callus. Means ± SD, n = 3, *** *p* < 0.001. (**E**) The expression pattern analysis of *PsbHLH25* in different tissues. Different letters indicated significant differences, Student’s *t* test, *p* < 0.05, means ± SD, n = 3.

To elucidate the molecular mechanism of *PsEXPA4-1* in response to exogenous GA_3_ signals to regulate the opening process of tree peony flowers, yeast one-hybrid was used to screen for upstream regulatory factors through the tree peony Yeast Library. Based on the screening results and the potential role in flower opening regulation, *PsbHLH25* was selected as the key upstream transcription factor of *PsEXPA4-1* for interaction verification and functional research. The yeast one-hybrid results revealed that PsbHLH25 could bind to the *PsEXPA4-1* promoter to regulate its expression (Figure 7A). The dual-luciferase assay revealed that PsbHLH25 could upregulate the expression of *PsEXPA4-1* (Figure 7B). These results indicated that PsbHLH25 could bind and promote the transcriptional accumulation of *PsEXPA4-1*. To investigate the potential role of *PsbHLH25* in the flower opening process of tree peony, we identified it from the transcriptome data. Expression pattern analysis revealed that treatment with 800 mg/L exogenous GA_3_ significantly promoted *PsbHLH25* expression (Figure 7C). Promoter activity analysis revealed that the *PsbHLH25* promoter is sensitive to exogenous GA signal in callus, indicating that *PsbHLH25* expression is regulated by exogenous GA signal (Figure 7D). Tissue-specific analysis revealed that *PsbHLH25* expression was the highest in petals (Figure 7E).

## 4. Discussion

Expansins are well-established regulators of cell wall loosening and have been implicated in flower opening in various plant species [7,8]. However, in tree peony the EXP gene family has remained uncharacterized, and the molecular mechanisms linking phytohormone signaling to petal expansion are largely unknown.

This study presents the genome-wide identification and analysis of the EXP gene family in tree peony, resulting in the identification of 36 members. The number of EXP genes is similar to that of *Arabidopsis* (36) but is lower than that of rice (*Oryza sativa*) (58) [33]. Phylogenetic analysis classified these genes into the following four subfamilies: EXPA, EXPB, EXLA, and EXLB. This classification is consistent with findings in model plants such as *Arabidopsis* and sweet potato (*Ipomoea batatas*), indicating a high degree of evolutionary conservation of the EXP gene family across the plant kingdom [33,34]. Notably, the EXPA subfamily contained the highest number of members (30), suggesting that *EXPA* genes may play a more extensive and crucial role in tree peony growth and development, particularly in processes like petal cell expansion. This observation aligns with studies in other ornamental species like petunia and rose, where the EXPA subfamily has also been identified as the primary regulator of petal cell expansion and flower opening [13,16]. Gene structure analysis revealed that members within the same subfamily share similar exon–intron patterns. This structural conservation is often linked to functional relatedness, implying that the tree peony EXP subfamilies may retain fundamental functions observed in other plants [33]. Further synteny analysis between tree peony and *Ar abidopsis* identified homologous blocks between some *PsEXP* and *AtEXP* genes. This microsynteny provides a reliable evolutionary basis for inferring the functions of *PsEXP* genes by leveraging knowledge from model plants.

GA signaling plays a crucial role in regulating flowering time in tree peony. On the one hand, GA can accelerate dormancy release in tree peony and may even substitute for the requirement of environmental chilling during the dormancy release process [26,35]. On the other hand, GA signaling also plays a significant regulatory role in the flower opening process, effectively accelerating the progression of flower opening in tree peony [28]. Analysis of cis-acting elements in the promoters showed that many *PsEXP* gene promoters are enriched with hormone-responsive elements, with GA-responsive elements being particularly prominent. Additionally, exogenous GA treatment specifically induced significant upregulation of eight genes, including *PsEXPA4-1*. Those results indicated that *PsEXP* genes are potentially involved in GA-mediated regulation of flowering time in tree peony. The promotive effect of *EXP* genes on GA-mediated regulation of flowering time has also been well-documented in various ornamental plants, including cabbage (*Brassica rapa*) [36] and rapeseed [22]. The subcellular localization of PsEXPA4-1 to the cell wall aligns perfectly with the established function of expansins, further supporting the conserved physiological pathway in tree peony wherein GA signaling promotes petal epidermal cell expansion and subsequent flower opening by upregulating *EXP* gene expression.

Further molecular and cellular characterization revealed that PsEXPA4-1 encodes a typical EXPA protein containing characteristic domains, and overexpressing *PsEXPA4-1* significantly promoted bolting and increased petal width in *Arabidopsis*. This phenotype is consistent with the *EXP* gene function reported in other plants. In *Osmanthus fragrans*, overexpression of *OfEXPA2* and *OfEXPA4* in *P. hybrida* both promoted the plant stem diameter and flower morphology [8]. *CmEXPA7* played a crucial role in altering petal cell size in chrysanthemum (*Chrysanthemum × morifolium*) [37]. In *Eustoma grandiflorum*, overexpression of *EgEXPA2* and *EgEXPA3* also resulted in petal growth and early flowering [38]. Although *EXPs* are broadly associated with growth and cell expansion processes across multiple plant tissues and our data suggested that *PsEXPA4-1* may play a role in flower opening, we cannot determine whether this role is specific to flower opening or reflects a more general function in GA-mediated growth responses. Future studies examining the tissue-specific effects of *PsEXPA4-1* manipulation will be required to address this question. Furthermore, it remains unclear how GA directly regulates the expression of *EXP* genes. The EXP expression can be regulated by changes in exogenous GA signal through numerous upstream transcription factors and participates in the regulation of plant growth and development. NAC transcription factors participated in GA-mediated endosperm expansion and seed germination by regulating *EXPA2* expression. RGL2 can interact with the NAC25 protein and inhibited the activity of the *EXPA2* promoter [39]. The study of banana (*Musa acuminata*) revealed that *MabHLH7* activated the promoters of cell wall modification genes, such as *MaEXP2/21*, and regulated the ripening process of banana fruits [40]. Our study identified a bHLH-class transcription factor, PsbHLH25, which was strongly induced by GA and highly expressed in petals. It could directly bind to and activate the *PsEXPA4-1* promoter. Our findings suggested a working model in which GA signaling promotes flower opening in tree peony through the PsbHLH25-PsEXPA4-1 module. The bHLH transcription factor family plays extensive roles in plant development and hormone responses [41]. In chrysanthemum, the bHLH transcription factor CmBPE2 could interact with CmJAZ1-like to regulate petal size [37]. In *O. fragrans*, the bHLH protein OfBPEub interacted with OfARF6 to activate the expression of *OfEXP15-4* to control flower opening process [42]. The bHLH transcription factor SlUPA-like in tomato (*Solanum lycopersicum*) was a key integrator of GA signaling to regulate the plant development [43]. However, the proposed GA-PsbHLH25-PsEXPA4-1 module has not been fully established due to the lack of stable transformation in tree peony, and our future research will focus on validating the necessity of *PsEXPA4-1* and *PsbHLH25* and exploring the upstream regulatory mechanisms of *PsbHLH25*. Furthermore, while our data showed that GA treatment induced *PsbHLH25* expression and the *PsbHLH25* promoter was GA-responsive, a direct causal relationship between GA signaling and *PsbHLH25* regulation has not been fully established. It remains possible that additional intermediate factors mediate this response.

In conclusion, this study systematically characterizes the EXP gene family in tree peony and establishes *PsEXPA4-1* as a key effector downstream of GA promoting flower opening. In addition, we discovered a novel transcriptional activator, *PsbHLH25*, connecting GA signaling to *PsEXPA4-1*, providing a testable hypothesis for future investigation. These results advance the molecular mechanistic research on tree peony flower opening, which will contribute to mapping a more comprehensive regulatory network for tree peony flower opening regulation and provide a solid theoretical foundation for the industry’s development.

## Data Availability

The original contributions presented in the study are included in the article/Supplementary Material, further inquiries can be directed to the corresponding authors.

## Supplementary Materials

The following supporting information can be downloaded at https://www.mdpi.com/article/10.3390/genes17050586/s1, Figure S1: The expression analysis of *PsEXPs* under different treatment conditions. Different letters indicate significant differences, Mean ± SD, n = 3, *p* < 0.05; Figure S2: Sequence alignment and phylogenetic tree analysis of PsEXPA4-1 protein. (A) The amino acid sequence alignment of EXPs from different species. Conservative areas were marked with red rectangular boxes. Sequences from *Carica papaya* (*CpEXPA8*, XP_021904225.1), *Morus notabilis* (*MnEXPA4*, XP_010108480.1), *Quillaja saponaria* (*QsEXPA4*, KAJ7964314.1), *Fagus crenata* (*FcEXPA8*, GMY10440.1), *Spatholobus suberectus* (*SsEXPA8*, TKY56599.1), *Sesamum indicum* (*SiEXPA4*, XP_011089527.1) and *Actinidia eriantha* (*AeEXPA4*, XP_057513338.1) were downloaded from GenBank. (B) Phylogenetic tree analysis of PsEXPA4-1 and EXPs from other species; Figure S3: The length analysis of the petals in transgenic plants. Student’s *t* test, *p* < 0.05. Means ± SD, n = 3; Table S1: The primer sequences used in this study; Table S2: The collinearity analysis between EXP in tree peony and *Arabidopsis*.
