# Supplementary material for: Identification of the PsEXP Gene Family and Functional Analysis of PsEXPA4-1 During Flower Opening Process in Tree Peony (Paeonia suffruticosa)
Source: Genes (Basel). 2026 May 21;17(5):586. doi: 10.3390/genes17050586 (PMC13205457; doi:10.3390/genes17050586)
Supplement: Supplementary file 1 [file genes-17-00586-s001.zip › Supplementary Files.pdf]

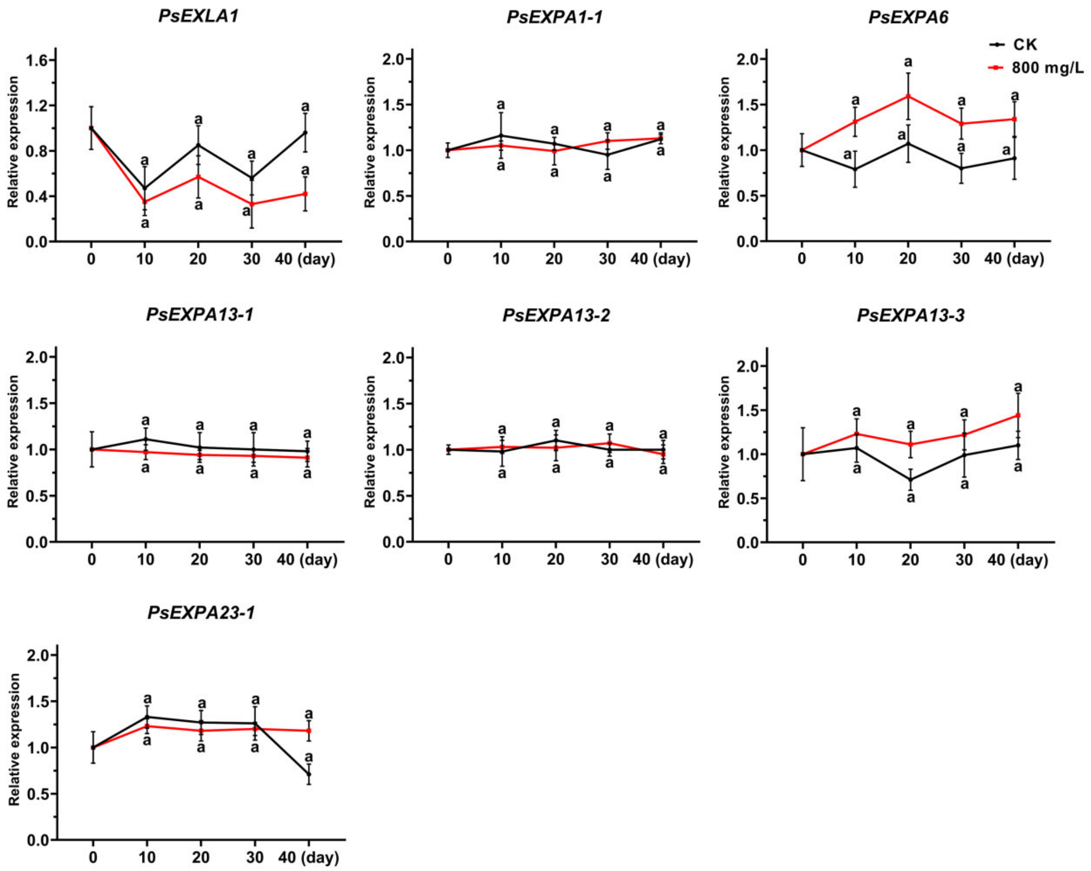

Supplementary Figure S1 The expression analysis of *PsEXPs* under different treatment conditions. Different letters indicate significant differences, Mean ± SD, n = 3, $P < 0.05$.

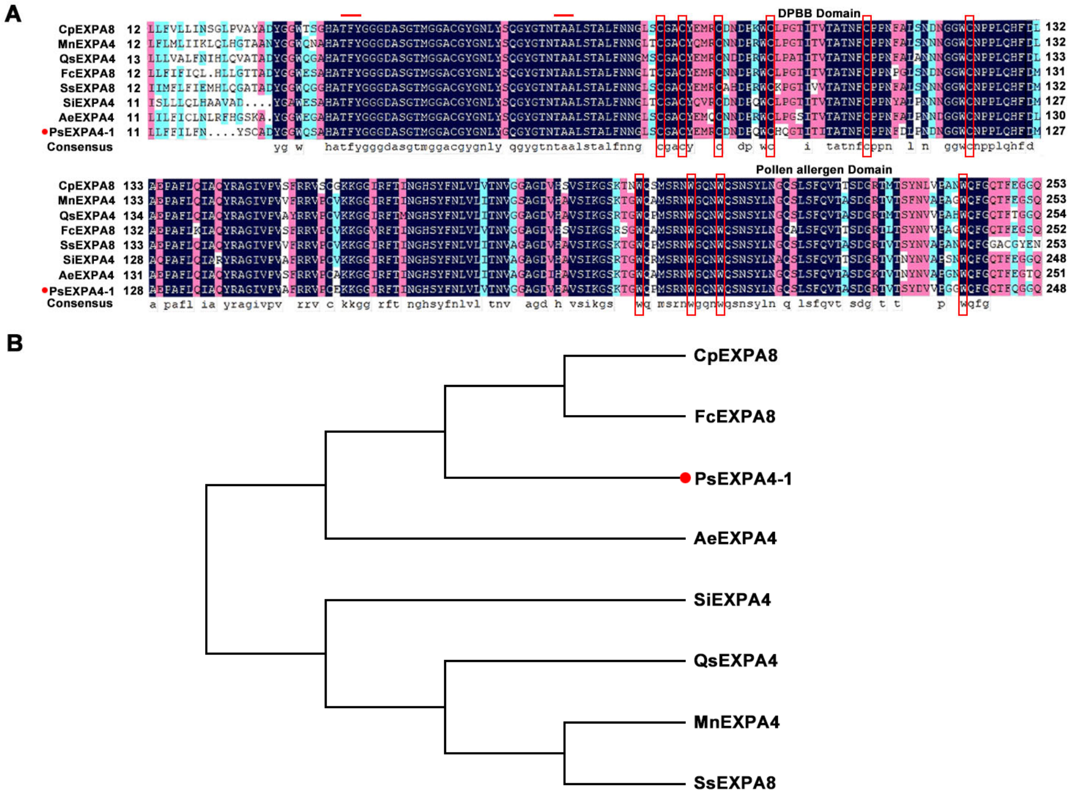

Supplementary Figure S2 Sequence alignment and phylogenetic tree analysis of PsEXPA4-1 protein. (A) The amino acid sequence alignment of EXPs from different species. Conservative areas were marked with red rectangular boxes. Sequences from *Carica papaya* (*CpEXPA8*, XP_021904225.1), *Morus notabilis* (*MnEXPA4*, XP_010108480.1), *Quillaja saponaria* (*QsEXPA4*, KAJ7964314.1), *Fagus crenata* (*FcEXPA8*, GMY10440.1), *Spatholobus suberectus* (*SsEXPA8*, TKY56599.1), *Sesamum indicum* (*SiEXPA4*, XP_011089527.1) and *Actinidia eriantha* (*AeEXPA4*, XP_057513338.1) were downloaded from GenBank. (**B**) Phylogenetic tree analysis of PsEXPA4-1 and EXPs from other species.

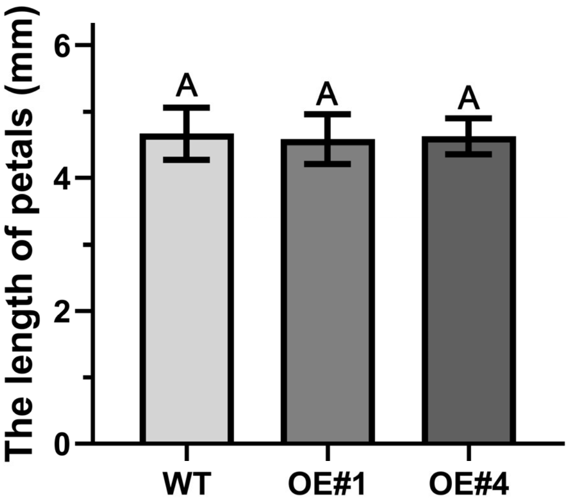

Supplementary Figure S3 The length analysis of the petals in transgenic plants. Student's t test, $P < 0.05$. Means ± SD, n = 3.
